# Transarterial Chemoembolization Combined With Tyrosine Kinase Inhibitors for Intermediate‐Stage Hepatocellular Carcinoma, What Else Can We Do?

**DOI:** 10.3389/fonc.2022.824799

**Published:** 2022-03-29

**Authors:** Jun Deng, Feng Wen

**Affiliations:** Department of Radiology, Shengjing Hospital of China Medical University, Shenyang, China

**Keywords:** hepatocellular carcinoma, intermediate stage, tyrosine kinase inhibitors, transarterial chemoembolization, combination therapy

## Abstract

Transarterial chemoembolization (TACE) has been considered the standard treatment for intermediate-stage hepatocellular carcinoma (HCC). However, intermediate‐stage HCC is highly heterogeneous with a broad population with varying tumour burdens, liver function. This suggests that TACE monotherapy treatment might not be suitable for all patients with intermediate‐stage HCC. The administration of tyrosine kinase inhibitors (TKIs) has become an important treatment option for improving the prognosis of patients with advanced HCC. Over the years, several trials have been conducted to explore the effects of TACE combined with TKIs for intermediate-stage HCC. However, the clinical efficacy is still controversial, and its potential clinical utility needs to be confirmed. This review will focus on the recent progress of TACE combined TKIs for intermediate-stage HCC.

## Introduction

Hepatocellular carcinoma (HCC) is the fourth leading cause of cancer-related deaths worldwide, and the prognosis of unresectable HCC is poor (1, 2). Chronic liver disease caused by hepatitis B and C viral infections is an important pathogenic factor for HCC (3, 4). However, with the anti-viral treatment in recent years, most HCC patients developed from hepatitis virus infection have decreased. In addition, non-alcoholic fatty liver disease (NAFLD) has gradually become prominent with increasing numbers of patients with diabetes mellitus, obesity, and hyperlipidemia (5–8). Approximately 20-30% of patients with NAFLD develop non-alcoholic steatohepatitis (NASH), and 10-20% of that develop cirrhosis (9, 10). Additional HCC patients are expected worldwide with the advances in surveillance programs and early diagnosis. The patients with intermediate-stage HCC do not often benefit from the transarterial chemoembolization (TACE) procedure due to its heterogeneity (11, 12). More and more physicians realize the importance of intermediate-stage HCC substaging. According to the 2022 Barcelona Clinic Liver Cancer (BCLC) version stratifies, TACE is only suitable for patients with well-defined nodules, preserved portal flow, and selective access (13). In addition, incomplete TACE embolization can induce the overproduction of vascular endothelial growth factor (VEGF) and platelet-derived growth factor (PDGF), which may promote tumor recurrence or metastasis (14, 15). Since most HCC patients have typically developed advanced stages with inferior prognosis, it is essential to prolong the patient’s duration in the intermediate‐stage HCC. The Food and Drug Administration (FDA) approves tyrosine kinase inhibitors (TKIs) for the treatment of advanced HCC because they can suppress tumor angiogenesis *via* the inhibition of multiple receptor (16). Recently, the combination of TACE with TKIs, such as sorafenib, has been confirmed to be a feasible and safe treatment (17–19). This review will attempt to analyze the present status of TACE combined TKIs for intermediate-stage HCC.

## TACE

The operative approach of conventional-TACE (cTACE) is to infuse a chemotherapy agent and lipiodol emulsions into the tumor-feeding arteries through a catheter under the guidance of medical imaging technology, followed by an injection of gelatin sponge particles to embolize the blood vessels (20). There are studies indicating that cTACE may significantly prolong survival in cases of intermediate-stage HCC compared with supportive care (21, 22). Some studies have shown that patients who respond to cTACE have a better prognosis and long-term survival (23, 24).

Although cTACE has been proven to have survival benefits for patients with intermediate-stage HCC, no optimal technique has been established (25). Due to the operator’s instability, the patient’s prognosis may also differ to a certain extent (26, 27). Drug-eluting beads transarterial chemoembolization (DEB-TACE) is to load chemotherapy drugs onto drug-loaded microsphere and then deliver them to the feeding artery of the hepatic tumor (28). This technology can achieve the sustained release of the chemotherapy drugs in the local tumor and reduce systemic exposure (29–31). Compared with the intra-arterial injection of chemotherapeutic drugs with or without lipiodol, DEB-TACE significantly reduces plasma concentrations of chemotherapeutic agents (32). Meanwhile, a previous study investigated serum VEGF level response after TACE with different embolic agents in patients with HCC and reported cTACE group had a more extraordinary rise in the circulating plasma levels of VEGF compared to the DEB-TACE group for 24-hour post-TACE and during the 4-week follow-up (114% vs. 164%, p=0.01; 123% vs. 170%, p=0.03) (33). This result indicates that DEB-TACE may better control tumors’ local recurrence and metastasis. However, many studies have compared the effectiveness of cTACE and DEB-TACE, and the results show that there is no statistical difference in the median overall survival (mOS) (34–37). For adverse events, DEB-TACE does not seem to perform better than cTACE. In the PRESCISION V study, there is no statistical difference (p=0.86) between cTACE (19.4%) and DEB-TACE (20.4%) in serious adverse events within 30 days after TACE (34). Recently, Zhang et al. showed that DEB-TACE caused more hepatobiliary injuries and severe abdominal pain (38).

Different sizes of DEBs may also influence the therapeutic effect of HCC patients. There are currently numerous bead sizes for clinical use. Some studies demonstrated that smaller DEBs enable more distal embolization, greater penetration, and tumor necrosis (39–41). Previously, multiple studies have demonstrated the effectiveness and safety of small-size DEBs for HCC patients, indicating that small-size DEBs have better application prospects for HCC patients (42–47).

The current clinical evidence was not sufficient to prove the superiority of DEB-TACE over cTACE. Thus, more high-quality clinical studies are certainly needed. The development of DEBs and the update of embolization technology also provide new options for the local treatment of intermediate-stage HCC.

## TKIs

Most HCC nodules are supplied by the hepatic artery. Angiogenesis plays a vital role in tumor occurrence, development, invasion, and metastasis (48). Angiogenesis of HCC is predominantly related to the out-of-control information transmission of cells in the tumor. The main pathway included epidermal growth factor receptor (EGFR), platelet-derived growth factor receptor (PDGFR), vascular endothelial growth factor receptor (VEGFR), HGF/C-Met and fibroblast growth factor receptor (FGFR). These receptors’ activation further triggers the cascade of intracellular RAS/RAF/MEK/ERK protein kinase signaling, leading to an imbalance between pro and anti-angiogenesis (4, 49). In an animal study, researchers prepared the iodine 124-labeled iodoazomycin galactopyranoside as a PET tracer for imaging and found that the oxygen content in the tumor was significantly lower than that of normal liver cells in the mouse (50). This finding may indicate that liver tumor cells are in a hypoxic microenvironment, and hypoxia can strongly stimulate tumor angiogenesis (51–53). The generated abnormal tumor blood vessels can interfere with the treatment of HCC. Therefore, we can improve the treatment efficacy of HCC through improving hypoxic microenvironment of tumor cells and normalizing the tumor vasculature. VEGF is widely considered an essential regulator of HCC tumor-induced angiogenesis. Overexpression of VEGF can cause uneven blood flow distribution and oxygen delivery in tumor blood vessels (54, 55). TKIs drugs can act on different kinase receptors. For example, sorafenib, which was first approved for the treatment of advanced HCC, can act on receptors such as VEGFR1-3, PDGFR-β, C-kit, RET, and PLT3, and extending the survival of patients with advanced HCC by blocking the information transmission of tumor cells, inhibiting tumor angiogenesis, promoting the normalization of tumor blood vessels (56, 57).

## Intermediate‐Stage HCC (BCLC Stage B)

Intermediate‐stage HCC is highly heterogeneous with a broad population. The differences were mainly reflected in the clinical characteristics, liver function, performance status, and tumor burden. For long times, TACE has been the standard and effective therapy for intermediate-stage HCC. However, this treatment is not suitable for all patients with intermediate-stage HCC (12). TACE is suitable for some patients with a small tumor burden and well-preserved liver function (58, 59). Previous randomized studies have shown that in selected patients with good liver function, the three-year survival rate of the TACE group is only 30% (60). Many patients require repeated TACE treatment because of incomplete embolization, which may deteriorate hepatic function and poor outcomes (61, 62).

The screening and stratification of the suitable population for TACE is essential. Some studies have performed the subclassification of the intermediate-stage group and the design of treatment strategies. In 2012, a panel of experts first divided stage B HCC patients into stages B1-B4 and proposed the “beyond Milan” and the “within up-to-7” to guide clinical practice (63). A study by Ha et al. conducted a survival analysis and evaluation of this subclassification system with additional improvements in which B3 and B4 subclasses were merged as BIII. There are significant differences in the mOS of the three subclassifications (41.0 vs. 22.1 vs. 16.6 months, p ≤ 0.001) (64). In 2016, Kudo et al. updated Bolondi’s subclassification modified for intermediate-stage HCC (Kinki Criteria). This subclassification divides intermediate-stage HCC into B1,B2,B3 mainly based on the Child-pugh score, beyond Milan and within up-to-7 (65). A subsequent study validated the Kinki Criteria and showed a statistically significant difference in mOS among the three substages (40.5 vs. 28.1 vs. 13.0 months, p ≤ 0.001) (66). The seven-eleven criteria proposed by Hung et al. recently divided intermediate-stage HCC into low tumor burden, intermediate tumor burden, and high tumor burden. The results show that this substage has significant discriminative power for mOS in three subgroups (33.1 vs. 22.3 vs. 11.9 months, p ≤ 0.001) (67). At present, many subclassifications of stage B HCC have been proposed, and several clinical studies have verified (68–73) (**Table 1**). The subclassification of BCLC stage B HCC is of significant value for the evaluation of patient prognosis as well as the selection of treatment protocols. Only patients who are suitable for TACE treatment can obtain the ideal survival benefit.

**Table 1:** Some substaging systems of intermediate stage HCC.

Criteria	Reference indicators	BCLC sub-stage	Number of patiens	mOS(months)	1st treatment option	Alternative treatment
Borondi et al. (63)*	CPT score Beyond MC and within Ut7 ECOG	B1 B2 B3 B4	101 232 35 98	41.0 22.1 14.1 17.2	TACE TACE or TARE - BSC	LT/TACE+ablation Sorafenib Research trials/TACE/sorafenib LT
Kudo et al, (65)	CPT score Beyond MC and within Ut7	B1 B2 B3:B3a B3b	158 236 31	46.8 30.0 13.2	LR/Ablation/Superselective cTACE DEB-TACE(>6cm)/HAIC(>6tumors)/Sorafenib (CP-A) LT/ Ablation /Superselective cTACE HAIC/Selective DEB-TACE	DEB-TACE (large, C-P 7) B-TACE cTACE DEB-TACE/B-TACE/HAIC BSC
Hung et al. (67)	7-11	Low TB Intermediate TB High TB	185 224 223	33.1 22.3 11.9	–	–
Yamakado et al. (68)	CPT score 4-of-7	B1 B2 B3	139 180 12	40.5 28.1 13.0	–	–
Hiroka et al. (71)	ALBI grade Beyond MC and within Ut7	B1 B2 B3 B4	94 175 452 33	63.5 38.1 28.0 12.5	LR/RFA/TACE RFA/TACE TACE/HAIC/sorafenib (CP-A) TACE/HAIC/BSC/LT	–
Hu et al. (72)	CPT socre Ut7	B1 B2 B3	165 671 190	29.0 19.0 10.0	TACE+LR.LT/RFA TACE TACE+systemic therapies	–
Kim et al. (73)	CPT socre Ut11 ECOG	B1 B2 B3	410 364 47	44.8 21.5 11.3	TACE TACE Sorafenib/HAIC	–

MC, Milan criteria; Ut7/Ut11, maximum tumor diameter plus tumor number less than 7/11; 7-11, the sum of maximum tumor diameter and tumor number; 4-of-7, the four tumors of 7 cm criterion; LR, liver resection; LT, liver transplantation; HAIC, hepatic artery infusion chemotherapy; BSC, best supportive care; RFA, radiofrequency ablation; *the OS date from a study by Ha et al. (64).

## Combination of TACE and TKIs

Although TACE is the standard treatment for intermediate‐stage HCC, TACE is unlikely to bring long-term clinical benefits to all patients with intermediate‐stage HCC. Furthermore, TACE causes the hypoxic microenvironment, leading to the upregulation of hypoxia-inducible factor-1α (HIF-1α). Increased HIF-1α then upregulates the expression of VEGF and PDGF and increases tumor angiogenesis (14, 15, 74). For intermediate‐stage HCC, TACE treatment needs to be further optimized to improve the response rate, protect liver function, and prolong survival. TKIs can act on multiple kinase receptors to block the information transmission of tumor cells and inhibit tumor angiogenesis. TACE monotherapy often fails to bring good clinical outcomes to patients. Since TKIs came into the treatment field of HCC, the clinical researches of TACE combined with TKIs for the treatment of intermediate‐stage HCC is continuously being explored and improved (**Table 2**).

**Table 2:** Some researches on TACE combined with TKIs.

Trail	Trail design	Type of trail	Inclusion criteria	TACE procedure	Definition of progression	Median OS/PFS/TTP months	The time of TKIs administration	Median medication time (weeks)	Median medication dose (mg/d)	limitations	Common AEs* (all grade/grade 3,4)	Liver toxicities (all grade/grade 3,4)
Post-TACE (18)	S+TACE vs. P+TACE	RCT, III	Child-Pugh A	cTACE,1-2 times	RECICL 2004	TTP (5.4 vs. 3.7, p = 0.252)	1-3 months after TACE	17.1	386	much lower than planned median daily dose of sorafenib (386 mg).	HFSR (82%35%)	Elevated AST (25%/12%)
	229 vs. 229		ECOG 0/1								Alopecia (41%/-)	Elevated ALT (21%/8%)
			BCLC B:n.a.								Rash (40%/4%)	
											Diarrhoea (31%/6%)	
											Hypertension (31%15%)	
BRISK-TA (75)	B+TACE vs. P+TACE	RCT, III	Child-Pugh A/B	cTACE/D-TACE,on-demand	mRECIST	OS (26.4 vs. 26.1, p =0.5280)	After TACE	24	n.a.	early closure.	D-appetite (82%35%)	Elevated AST (35%/14%)
	57 vs. 59		ECOG 0/1							differences by region.	Fatigue (43%/4%)	Elevated ALT (36%/10%)
			BCLC B:52%								hypertension (47%/14%)	
											A-pain (31%/6%)	
											Diarrhea (36%/7%)	
SPACE (76)	S+TACE vs. P+TACE	RCT, II	Child-Pugh A	DEB-TACE, on a fixed interval	mRECIST	TT P (5.6 vs. 5.5, p =0.072)	3-7 days before TACE	21	566	the restrictive definition of TTUP.	A-pain (60.1%/7.8%)	Elevated AST (24.8%/24.2%)
	154 vs. 153		ECOG 0							DEB-TACE on a fixed interval.	Diarrhea (52.9%/3.9%)	Elevated ALT (17.0%/15.1%)
											HFSR (46.4%/9.2%)	
			BCLC B:100%								Fatigue (43.1%/11.1%)	
											Fever (38.6%/0)	
TACE-2 (77)	S+TACE vs. P+TACE	RCT, III	Child-Pugh A	DEB-TACE on demand	RECIST1.1	PFS (7.7 vs. 7.8, p =0.94)	2-5 weeks before TACE	17	660	heterogeneity in baseline characteristics.	Fatigue (81%/19%)	n.a.
	139 vs. 138		ECOG 0/1							the strict criteria for retreatment.	A-pain (60%/13%)	
			BCLC B:n.a.								Diarrhoea (56%/11%)	
											Nausea (46%/1%)	
											Rash (38%/2%)	
ORIENTAL (78)	O+TACE vs. P+TACE	RCT, III	Child-Pugh A	cTACE, on demand	n.a.	OS (31.1 vs. 32.3, p =0.435)	3-28 days after TACE	43.6	n.a.	early termination.	A-pain (71%/6%)	Elevated AST (50%/43%)
	363 vs. 364		ECOG 0/1							heterogeneity in baseline characteristics.	Pyrexia (59%/<1%)	Elevated ALT (45%/34%)
			BCLC B:47%								D-appetite (47%/4%)	
											Constipation (40%/<1%)	
											Nausea (39%/<1%)	
TACTICS (79)	S+TACEV vs. TACE	RCT, II	Child-Pugh A/B7	cTACE, on demand	Inability to TACE/TACE failure/New Lesions are not considered as progress.	PFS (36.2 vs. 30.8; p =0.40) OS(22.8 vs. 13.5, p =0.02)	2-3 weeks before TACE	38.7	355.2	small number of patients included.	Anaemia (64.9%/1.3%)	Elevated AST (93.5%/28.6%)
	80 vs. 76		ECOG 0/1							modified unTACEable progression.	HFSR (53.2%/5.2%)	Elevated ALT (89.6%/24.7%)
			BCLC B:55%								Hypertension (51.9%/10.4%)	
											Malaise (26.0%/0)	
											Fatigue (24.7%/2.6%)	
Fu et al. (99)	L+TACE vs.	retrospective, nRCT	Child-Pugh A/B	D-TACE, on-demand	mRECIST	n.a.	3 days after TACE	n.a.	32.9	retrospective study.	Hypertension (48.3%/23.3%)	Elevated AST (38.3%/3.3%)
	TACE		BCLC B:55%							patients’ willing to choose the lenvatinib treatment.	Bleeding** (21.7%/1.7%)	Elevated ALT (23.3%/1.7%)
	60 vs. 60									small sample size.	Diarrhea (18.3%/0)	
											Diarrhea (16.7%/0)	
											Dysphonia (15.0%/0)	
Guo et al. (80)	A+TACE vs.	retrospective, nRCT	Child-Pugh A/B	cTACE, on demand	mRECIST	n.a.	3-5 days after TACE	n.a.	n.a.	retrospective study.	HFSR (47.2%/n.a)	n.a
	TACE		ECOG 0/1							small sample size.	Hypertension (27.8%/n.a)	
	60 vs. 60		BCLC B:44%							the short follow-up time.	Erythra (22.2%/n.a)	
											Proteinuria (13.9%/n.a)	
											Hypothyroidism (8.3%/n.a)	

n.a., not available; OS, overall survival; PFS, progression-free survival; TTP,tome-to-progression; L, Lenvatinib; A, anlotinib; S, sorafenib; P, placebo; O, orantinib; B, brivanib; RCT, randomized controlled trial; D-appet, decreased appetite; A-pain, abdominal pain; AST, aspartate aminotransferase; ALT, alanine aminotransferase; *Top five common AEs; **Bleeding (gingiva).

### Combination of TACE and Sorafenib

In phase I clinical study of TACE combined with sorafenib in the treatment of HCC, Dufour et al. confirmed that the adverse effects of the combination therapy are equivalent to those of sorafenib monotherapy. After the combination therapy, VEGF concentrations in serum decreased from 93 ng/l to 67 ng/l, and this suggests that the combined regimen may reduce the overexpression of VEGF in the blood and inhibit the recurrence and metastasis of tumors (19). The way of administration in this study may impact the outcome of the HCC patients, which uses continuous administration (i.e., dose-escalation, and without drug discontinuation post-TACE and pre-TACE). Kudo et al. reported a phase III multi-center randomized controlled study (Post-TACE) that included Korean and Japanese patients of TACE combined with sorafenib for unresectable HCC (18). Patients with an objective response after the last TACE were given oral sorafenib within 1-3 months based on their liver function. However, the final results of the Post-TACE trial showed no significant difference in time to progression (TTP) between combination and control groups, which may be related to the low therapeutic dose of sorafenib (386mg) of the combination therapy. In this study, 60% of patients have delayed administration for more than nine weeks before randomization. The peak of the VEGF concentration in the circulating blood reached on the first day after TACE (14), so the interval between sorafenib administration before and after TACE should not be too long. In the exploratory analysis of this study, it was found that Korean patients had a better TTP hazard ratio (HR, 0.38 vs. 0.94) compared with Japanese patients, which may be related to the longer median duration of sorafenib (31 weeks vs. 16 weeks).

At the same time, Llovet et al. carried out a phase II, randomized, double-blind clinical study (SPACE) (76). Sorafenib was administrated for pre-treatment 3-7 days before the first TACE in the combined therapy group to promote the normalization of tumor blood vessels. Time to untraceable progression (TTUP), as the secondary endpoint, was proposed for the first time in this study. It is defined as a nodule receiving treatment that fails to achieve objective response after at least two TACE treatments or has contraindications for chemotherapy regimens, including macrovascular invasion (VMI), extrahepatic spread (EHS), persistent ascites, and liver function Child-Pugh B grade or ECOG PS> 2 or platelet count ≤60×10^9^/L. However, no statistically significant difference was observed in TTP between the combination and the monotherapy group in this study, which may be related to the restrictive definition of TTUP. Because there will be transient liver function abnormalities and blood biochemical parameters change after TACE, it may be inappropriate to be defined as disease progression at this time. In addition, the TACE procedure in this study was performed at fixed intervals. When intrahepatic lesions respond well to TACE, unnecessary repeated TACE may impair liver function or increase the side effects of sorafenib (81). Although the primary endpoint of this trial was not statistically different, the hazard ratio of time to VMI/EHS between the combined therapy group and the monotherapy group was 0.621. This exploratory trial suggests that the combination of sorafenib plus DEB-TACE was feasible in patients with intermediate-stage HCC.

Phase III clinical study (TACE-2) of TACE combined with sorafenib conducted by Meyer et al. in a European population also showed negative results (77). No significant statistical difference between the combined therapy and the monotherapy groups were found in progression-free survival (PFS) (230 vs. 235 days, p=0.94). The failure of TACE-2 may be related to the definition of disease progression. The appearance of new lesions in the liver may not be a sign of stopping TACE or sorafenib treatment and switching to other treatment methods because it is the natural characteristic of HCC. Therefore, it may not be appropriate to use RECIST 1.1 or mRECIST evaluation criteria to define HCC progression after the combined therapy.

Based on these previous studies, a multi-center, randomized controlled, phase II study (TACTICS) confirmed the benefits of combination therapy (79). This study showed that the combined therapy group and monotherapy group had a statistical difference in the primary endpoint of PFS (25.2 months vs. 13.5 months; p=0.006). The secondary endpoints of the two groups, such as TTP (26.7 vs. 16.4 months, p=0.005), time to stage progression time (22.5 months vs. 6.3 months, p=0.001), were significantly different. The most outstanding innovation of this study was that the appearance of new lesions in the liver is not defined as tumor progression. However, the results of the TACTICS study updated in the latest ASCO GI meeting showed that no statistical difference was observed between the combined therapy group and monotherapy group in the median OS (36.2 months vs. 30.8 months; p=0.40). Updated PFS between the two groups is still significantly different (22.8 months vs. 13.5 months, p=0.02) (82).

The analysis found that the follow-up anti-tumor treatment of the trial was more common in the TACE monotherapy group (76.3% vs. 58.5%), and the administration of sorafenib treatment accounted for a higher proportion in the TACE monotherapy group (50% vs. 10.6%). This result might be because patients in the combined therapy group developed resistance to sorafenib treatment after progression. This follow-up positive anti-tumor and systemic therapy (i.e., radiofrequency ablation, TACE, hepatic artery infusion chemotherapy, or other targeted and immune drugs) prolonged survival after progression, and confounded survival analysis and diluted the OS benefit of the combined therapy group. The positive results of PFS in the TACTICS could be due to several reasons. First, new lesions in the liver were not considered tumor progression, which prolonged the combination therapy time. Second, the standard of TTUP is looser than that of the SPACE study. This also prolonged the time to change other treatment methods. The median average dose of sorafenib in this study was only 355.2mg, but the duration of the drug was long enough (38.7 weeks). As in the SPACE study, pre-treatment with 400mg sorafenib day was given before TACE to observe the patient’s tolerance to the drug and promote the normalization of tumor blood vessels. However, the pre-treatment time of TACTICS was longer for 2-3 weeks. At the same time, stopping medication is conducive to preserving liver function two days before and after each TACE. Therefore, for combined therapy of intermediate-stage HCC, we should try our best to protect liver function and extend the duration of drug medications, which may be more conducive to the survival of patients than the maximum dose of the drug. Although the OS in this trial was not statistically different between the combined therapy group and monotherapy group, the patients in the combined therapy group extended the time to stage progression, which allowed the patients to stay in the intermediate stage for a longer time and obtain a better quality of life.

For intermediate-stage HCC combination therapy studies, OS may not be a suitable primary endpoint. As a critical endpoint of cancer treatment research, OS has its limitations. First, it may require an extended follow-up to obtain sufficient patient data. Moreover, PFS seems to be a surrogate primary endpoint for OS. A study by Llovet et al. showed that the threshold of PFS ≤ 6 can predict the improvement of OS in advanced HCC (83). However, the benefits of PFS in the TACTICS had not been converted into the benefits of OS. The selection of appropriate endpoints for combination therapy is a question that still needs to be addressed in future clinical trials. Once patients are defined as disease progression during combination therapy, other treatment modalities must be introduced according to the clinical guidelines. However, whether the disease progression is the failure of combination therapy or the natural tumor biology of HCC still remains ambiguous. If the latter was the case, OS data might be confounded by follow-up treatment after disease progression (84). So the definition of progression may require refinement, especially in the combination therapy of the intermediate-stage HCC. The definition of disease progression affects TACE and sorafenib’s performance, thereby affecting the endpoints of the trial analysis. At present, more and more interventional physicians are beginning to consider that the appearance of new lesions in the liver cannot be counted as progress.

### Combination of TACE and Brivanib

Park et al. confirmed the efficacy of brivanib for advanced HCC (85). The study included 55 patients with unresectable, advanced, or locally metastatic HCC. Studies have confirmed that brivanib and sorafenib are equally effective in treating advanced HCC. The HCC patients is well tolerated with brivanib. Based on this phase II study results, Kudo et al. investigated brivanib as an adjuvant combination therapy for TACE (75). This randomized, double-blind, placebo-controlled phase III clinical study (BRISK-TA) enrolled a total of 870 HCC patients who met TACE criteria. After the first TACE, they were randomly assigned (1:1), and 800mg of brivanib and placebo were taken each day orally. The administration of brivanib in the study varied from 2 to 21 days after TACE according to liver function. There was no statistically significant difference in OS between the combined therapy and monotherapy groups (26.4 months vs. 26.1 months, p=0.5280).

Regarding the negative results of this study, Kudo et al. consider that the trial only recruited 502 patients due to early termination, which is less than the planned 870 patients (81). Although no positive results were observed in mOS, there were statistical differences between time to extrahepatic spread (TTES)/time to vascular invasion (TTVI) and objective response rate. The number of TACE procedures in the combined therapy group is also less than in the monotherapy group. All these indicated that TACE combined with brivanib has a positive anti-cancer effect.

### Combination of TACE and Orantinib

Orantinib is a multi-targeted, orally active, small-molecule tyrosine kinase inhibitor (TKI) that inhibits the VEGF-2 and the PDGF-β receptor (86, 87). Many clinical trials have confirmed the safety and effectiveness of orantinib in treating advanced HCC. In the study by Inaba et al., patients treated with TACE monotherapy were randomly divided into orantinib and no medication groups (88). A total of 103 patients were included in the study. The results showed that the median PFS of the combined therapy group and monotherapy group were 157 and 122 days, respectively. Although there was no statistical difference between the two groups, the mPFS of the combination group had a significant prolongation trend. It is necessary to test the combination of TACE and orantinib further. Kudo et al. explored the efficacy of TACE combined with orantinib in a randomized, double-blind, placebo-controlled, multi-center multi-center, phase III study (ORIENTAL) (78). A total of 889 patients were enrolled in this study. These patients were randomly assigned to the combined therapy and monotherapy groups at a 1:1 ratio. Orantinib administration was given 200mg orally twice a day and 3-28 days after TACE according to whether the patients met the criteria for administration. There was no statistically significant difference in mOS between the combination and control groups (31.1 months vs. 32.3 months, p=0.5280). In the subgroup analysis, it was found that Japanese patients observed a trend toward improved mOS compared with the control group. This could be due to better medication dosages control in Japanese patients. About 50% of Japanese patients had reduced their medication dosages, while only 25% of patients in Korea and Taiwan have reduced their dose. A timely reducing drug dose may decrease drug toxic side effects, affecting patients’ treatment and prognosis.

### Combination of TACE and Anotinib

A phase III randomized clinical study confirmed that anlotinib has survival benefits for non-small cell lung cancer (89). The mechanism action of anlotinib may be through the Erk and Akt pathways to inhibit HCC proliferation, suppress tumor growth, and induce tumor apoptosis (90–92). A retrospective study compared TACE combined with anlotinib and TACE monotherapy to treat intermediate-stage HCC (80). The study included 82 patients with unresectable HCC. Patients in the combined therapy group (n=36) took orally anlotinib 12 mg daily for 3-5 days after the first TACE (taken for two weeks and stopped for one week). The results demonstrated a significant difference in PFS (7.35 months vs. 5.54 months, p=0.035). Although no statistical difference was observed in the 3-month survival rate (97.2% vs. 93.5%, p=0.627), the 6-month and 1-year survival rate of the combined therapy group (83.3% vs. 56.5%, p=0.016; 66.7% vs. 19.6%, p = 0.016) are significantly higher than monotherapy group. Meanwhile, no grade 4 adverse events were observed in the two groups of patients, and all the adverse events were alleviated after treatment or dose adjustment. The follow-up durations in this study were relatively short. Whether the benefit of PFS translates into OS benefit is still unclear. Further researches, preferably with large clinical studies, are needed to confirm the clinical effect of TACE combined with anlotinib.

### Combination of TACE and Lenvatinib

Lenvatinib is a novel oral multi-kinase inhibitor that targets vascular endothelial growth factor (VEGF) receptors 1–3, fibroblast growth factor (FGF) receptors 1–4, platelet-derived growth factor (PDGF) receptor-alpha, rearranged during transfection (RET), and KIT (93–97). Recently, an open-label, multi-center phase III clinical randomized non-inferiority study (REFLECT) compared the efficacy of lenvatinib and sorafenib in patients with advanced HCC (98). The results demonstrated that most of the lenvatinib group was comparable to that of the sorafenib group. A recent retrospective study compared TACE combined with lenvatinib with TACE monotherapy to treat unresectable HCC (99). This study included 120 patients with unresectable HCC. Patients in the combination group took lenvatinib orally three days after TACE treatment and withdrew the drug three days before repeating on-demand TACE treatment. The dose of lenvatinib is mainly determined according to the weight of patients. Patients (bodyweight≥60kg) take 12mg, and patients (bodyweight<60kg) take 8mg. The final results showed that the combined therapy group’s 1-year and 2-year OS (88.4% and 79.8%) were higher than the control group’s (79.2% and 49.2%, P =0.047). In terms of PFS, the combination group was also better than the control group (1 year: 78.4% vs. 64.7%; 2 years: 45.5% vs. 38.0%, p<0.001). The combined therapy group also had a better objective response rate (68.3% vs. 31.7%, p<0.001). Meanwhile, the patients in the combined therapy group tolerated lenvatinib well. This study is the first retrospective study of TACE combined with lenvatinib in treating unresectable HCC. Although the results showed that the combined therapy group tends to prolong the OS and PFS, the median follow-up time of the combined therapy group and the control group is only 11.6 months and 17.5 months, respectively. The proportion of treatment in the combined group after disease progression is relatively lower (35.7% vs. 62.2%). The final results of OS and PFS is still unclear. In this study, the TACE treatment interval of the combined group was significantly longer than that of the control group (103.3vs.74.7d, p=0.004), which provided the possibility to protect the liver function of the patients. Therefore, the clinical efficacy of TACE combined with lenvatinib in patients may require further large-scale randomized controlled clinical studies to verify.

## What Else Can We Do?

The treatment of intermediate-stage HCC has always been a hotly debated topic. The emergence of molecule-targeted drugs has provided more treatment options for intermediate-stage HCC with TACE as the main therapeutic modality. With the emergence and update of various new drugs, researcher's attention and the pursuit of treatment effect for intermediate-stage HCC have also increased. The treatment goal of intermediate-stage HCC has gradually expanded from delaying disease progression to achieving tumour downstaging and undergoing curative conversion therapy. In the future, the exploration of treatment strategies for intermediate-stage HCC should focus on the prolongation of OS and the curative conversion therapy after tumour downstaging.

The advent of immune checkpoint inhibitors (ICIs) may provide new directions for the combination treatment of HCC. Previous studies (e.g., KEYNOTE-224 and KEYNOTE-240) have confirmed that pembrolizumab has favorable disease control and side effects for HCC patients previously treated with sorafenib (100, 101). Recently, significant progress has been achieved in a global, open-label, phase 3 trial (IMBRAVE 150). This study compared the clinical efficacy of atezolizumab (anti-PDL1 checkpoint inhibitor) plus bevacizumab(anti-VEGF) and sorafenib for unresectable HCC. Results of the study demonstrated that the mPFS of patients in the atezolizumab–bevacizumab group was significantly longer than that in the sorafenib group (6.8 vs. 4.3 months, <0.001) (102). In the latest ASCO GI 2021 meeting, the result showed that mOS was significantly longer in the atezolizumab–bevacizumab group than in the sorafenib group (19.2 vs. 13.4 months, <0.001). Therefore, in the 2022 updated BCLC strategy, the atezolizumab–bevacizumab therapy is recommended as the first-line treatment for advanced HCC (13). It is not difficult to see that the update of the treatment strategy for advanced HCC will bring more survival benefits to patients and affect the treatment strategy of intermediate-stage HCC. The earlier application of TKIs and their combination with TACE in intermediate-stage HCC could make it possible to reduce the number of TACE treatments, maximize the protection of liver function, and ultimately prolong the overall survival of patients with HCC.

Some studies of TACE combined with ICIs are on the way, and it is unclear whether this combination is beneficial for intermediate-stage HCC. However, a retrospective study by Zheng et al. demonstrated the safety and efficacy of TACE combined with sorafenib plus immune checkpoint inhibitors (TACE+Sor+ICIs) (103). This study included 51 patients with intermediate and advanced TACE-resistant HCC, divided into TACE+Sor+ICIs and TACE combined with sorafenib (TACE+Sor) groups. The results showed that the disease control rate of the TACE+Sor+ICIs group was significantly higher than that of the TACE+Sor group (81.82 vs. 55.17%, P = 0.046). Besides, they observed that the mPFS (16.26 vs. 7.30 months, P < 0.001) and mOS (23.3 vs. 13.8 months, P = 0.012) of the TACE+Sor+ICIs group was significantly longer than that of the TACE+Sor group. Another study also confirmed that for intermediate and advanced HCC, tumors in the TACE with molecular targeted agents (MTGs) plus immune checkpoint inhibitors (ICIs) group had a higher liquefactive necrosis rate than tumors in the TACE with MTGs group (30% vs. 4.8%, P=0.006) (104). If TACE is combined with TKIs plus ICIs in treating patients with intermediate-stage HCC, is it possible to acquire better clinical efficacy? This needs to be confirmed by further large clinical studies.

Based on the outcomes of REFLECT, lenvatinib is now approved for the first-line treatment of advanced HCC. In the REFLECT study, masked independent imaging review confirmed a significantly higher objective response rate in the lenvatinib arm than in the sorafenib arm by mRECIST (40.6 vs 12.4%, p<0.0001) (98). Previous studies have shown that ORR and sustained response duration are effective predictors of longer OS, and early treatment response remains a reliable predictor of a good prognosis (23, 24). At present, studies have explored how to translate the high objective response rate of lenvatinib into more prolonged survival in patients with intermediate-stage liver cancer. A study conducted by Kudo et al. demonstrated that lenvatinib has higher ORR (73.3% vs. 33.3%, p<0.001) and mOS (37.9 vs. 21.3 months, p<0.01) as first-line versus TACE for intermediate-stage HCC beyond up-to-seven Criteria and child-pugh A liver function (59). Another study investigated lenvatinib-TACE sequential therapy versus lenvatinib alone in patients with intermediate-stage HCC who were not unsuitable for TACE. The results showed that the OS of the combined treatment group was significantly longer than that of the lenvatinib group (not reached vs. 16.9 months, p = 0.007) (105). Two studies suggest that early lenvatinib-TACE sequential therapy may be a good combination therapy for patients with intermediate-stage HCC who are not suitable for TACE. Not just the ongoing TACTICS-Lenvatinib study, more randomized controlled trials are needed to confirm the clinical benefit of this combination in the intermediate-stage HCC. Not only that, but the high objective response rate of lenvatinib will also provide more opportunities for the transformation therapy of intermediate-stage HCC. It can be seen that lenvatinib has shown a trend of replacing other TKI drugs in the combined treatment of intermediate-stage HCC.

The extensive randomized controlled clinical studies of TACE combined with TKIs in the treatment of intermediate-stage HCC have all failed. In the future, the treatment of the intermediate-stage HCC remains challenging. The etiology of HCC gradually changes, and non-viral hepatitis caused by NAFLD and NASH increases. This may also change the holistic treatment concept of HCC in the future. It can be found that the combined treatment has survival benefits in specific subgroups of HCC patients. Therefore, substaging and guidelines for stage B HCC require more refined definitions. The combination treatment regimen for HCC patients should be individualized based on individual patient factors. The selection of the patient population for combination therapy will be very worthy of attention in the future. On the other hand, TKIs combined with more embolization treatments including cTACE, DEB-TACE and TARE need to be explored. At the same time, the efficiency improvement of TACE combined with TKIs might ultimately be implemented by improvement of embolization efficacy and technical limitations of TACE, preservation of liver function and management of adverse events. Several clinical trials are currently underway to explore the efficacy of combination therapy for intermediate-stage HCC. Therefore, better results can be expected in the future.

In conclusion, the road of combined therapy for intermediate-stage HCC is not smooth. However, combined therapy is an inevitable trend for the future development of HCC. It is believed that more optimized combination methods will bring more excellent clinical effects soon.

## Author Contributions

JD and FW wrote the manuscript. All authors contributed to the article and approved the submitted version.

## Funding

This work was financially supported by Beijing iGandan Foundation of China (GDXZ-08-10).

## Conflict of Interest

The authors declare that the research was conducted in the absence of any commercial or financial relationships that could be construed as a potential conflict of interest.

## Publisher’s Note

All claims expressed in this article are solely those of the authors and do not necessarily represent those of their affiliated organizations, or those of the publisher, the editors and the reviewers. Any product that may be evaluated in this article, or claim that may be made by its manufacturer, is not guaranteed or endorsed by the publisher.

## References

[1] Bray F Ferlay J Soerjomataram I Siegel RL Torre LA Jemal A. Global Cancer Statistics 2018: GLOBOCAN Estimates of Incidence and Mortality Worldwide for 36 Cancers in 185 Countries. CA: Cancer J Clin (2018) 68(6):394–424. doi: 10.3322/caac.21492 30207593

[2] Llovet JM Brú C Bruix J. Prognosis of Hepatocellular Carcinoma: The BCLC Staging Classification. Semin Liver Dis (1999) 19(3):329–38. doi: 10.1055/s-2007-1007122 10518312

[3] Chang MH You SL Chen CJ Liu CJ Lee CM Lin SM. Decreased Incidence of Hepatocellular Carcinoma in Hepatitis B Vaccinees: A 20-Year Follow-Up Study. J Natl Cancer Instit (2009) 101(19):1348–55. doi: 10.1093/jnci/djp288 19759364

[4] Villanueva A. Hepatocellular Carcinoma. N Engl J Med (2019) 380(15):1450–62. doi: 10.1056/NEJMra1713263 30970190

[5] McGlynn KA Petrick JL El-Serag HB. Epidemiology of Hepatocellular Carcinoma. Hepatol (Baltimore Md) (2021) 73(Suppl 1):4–13. doi: 10.1002/hep.31288 PMC7577946 32319693

[6] Huang DQ El-Serag HB Loomba R. Global Epidemiology of NAFLD-Related HCC: Trends, Predictions, Risk Factors and Prevention. Nat Rev Gastroenterol Hepatol (2021) 18(4):223–38. doi: 10.1038/s41575-020-00381-6 PMC8016738 33349658

[7] Younossi ZM Koenig AB Abdelatif D Fazel Y Henry L Wymer M. Global Epidemiology of Nonalcoholic Fatty Liver Disease-Meta-Analytic Assessment of Prevalence, Incidence, and Outcomes. Hepatol (Baltimore Md) (2016) 64(1):73–84. doi: 10.1002/hep.28431 26707365

[8] Estes C Anstee QM Arias-Loste MT Bantel H Bellentani S Caballeria J. Modeling NAFLD Disease Burden in China, France, Germany, Italy, Japan, Spain, United Kingdom, and United States for the Period 2016-2030. J Hepatol (2018) 69(4):896–904. doi: 10.1016/j.jhep.2018.05.036 29886156

[9] Ahmed A Wong RJ Harrison SA. Nonalcoholic Fatty Liver Disease Review: Diagnosis, Treatment, and Outcomes. Clin Gastroenterol Hepatol (2015) 13(12):2062–70. doi: 10.1016/j.cgh.2015.07.029 26226097

[10] Vernon G Baranova A Younossi ZM. Systematic Review: The Epidemiology and Natural History of Non-Alcoholic Fatty Liver Disease and Non-Alcoholic Steatohepatitis in Adults. Aliment Pharmacol Ther (2011) 34(3):274–85. doi: 10.1111/j.1365-2036.2011.04724.x 21623852

[11] Kudo M. A New Treatment Option for Intermediate-Stage Hepatocellular Carcinoma With High Tumor Burden: Initial Lenvatinib Therapy With Subsequent Selective TACE. Liver Cancer (2019) 8(5):299–311. doi: 10.1159/000502905 31768341 PMC6872999

[12] Kudo M Han KH Ye SL Zhou J Huang YH Lin SM. A Changing Paradigm for the Treatment of Intermediate-Stage Hepatocellular Carcinoma: Asia-Pacific Primary Liver Cancer Expert Consensus Statements. Liver Cancer (2020) 9(3):245–60. doi: 10.1159/000507370 PMC7325125 32647629

[13] Reig M Forner A Rimola J Ferrer-Fàbrega J Burrel M Garcia-Criado Á. BCLC Strategy for Prognosis Prediction and Treatment Recommendation: The 2022 Update. J Hepatol (2022) 76(3):681–93. doi: 10.1016/j.jhep.2021.11.018 PMC8866082 34801630

[14] Li X Feng GS Zheng CS Zhuo CK Liu X. Expression of Plasma Vascular Endothelial Growth Factor in Patients With Hepatocellular Carcinoma and Effect of Transcatheter Arterial Chemoembolization Therapy on Plasma Vascular Endothelial Growth Factor Level. World J Gastroenterol (2004) 10(19):2878–82. doi: 10.3748/wjg.v10.i19.2878 PMC4572123 15334691

[15] Wang B Xu H Gao ZQ Ning HF Sun YQ Cao GW. Increased Expression of Vascular Endothelial Growth Factor in Hepatocellular Carcinoma After Transcatheter Arterial Chemoembolization. Acta Radiol (Stockholm Sweden: 1987) (2008) 49(5):523–9. doi: 10.1080/02841850801958890 18568538

[16] Arora A Scholar EM. Role of Tyrosine Kinase Inhibitors in Cancer Therapy. J Pharmacol Exp Ther (2005) 315(3):971–9. doi: 10.1124/jpet.105.084145 16002463

[17] Jin PP Shao SY Wu WT Zhao XY Huang BF Fu QH. Combination of Transarterial Chemoembolization and Sorafenib Improves Outcomes of Unresectable Hepatocellular Carcinoma: An Updated Systematic Review and Meta-Analysis. Japanese J Clin Oncol (2018) 48(12):1058–69. doi: 10.1093/jjco/hyy138 30272196

[18] Kudo M Imanaka K Chida N Nakachi K Tak WY Takayama T. Phase III Study of Sorafenib After Transarterial Chemoembolisation in Japanese and Korean Patients With Unresectable Hepatocellular Carcinoma. Eur J Cancer (Oxford England: 1990) (2011) 47(14):2117–27. doi: 10.1016/j.ejca.2011.05.007 21664811

[19] Dufour JF Hoppe H Heim MH Helbling B Maurhofer O Szucs-Farkas Z. Continuous Administration of Sorafenib in Combination With Transarterial Chemoembolization in Patients With Hepatocellular Carcinoma: Results of a Phase I Study. Oncol (2010) 15(11):1198–204. doi: 10.1634/theoncologist.2010-0180 PMC3227910 21036880

[20] Lencioni R. Loco-Regional Treatment of Hepatocellular Carcinoma. Hepatol (Baltimore Md) (2010) 52(2):762–73. doi: 10.1002/hep.23725 20564355

[21] Llovet JM Real MI Montaña X Planas R Coll S Aponte J. Arterial Embolisation or Chemoembolisation Versus Symptomatic Treatment in Patients With Unresectable Hepatocellular Carcinoma: A Randomised Controlled Trial. Lancet (London England) (2002) 359(9319):1734–9. doi: 10.1016/s0140-6736(02)08649-x 12049862

[22] Cammà C Schepis F Orlando A Albanese M Shahied L Trevisani F. Transarterial Chemoembolization for Unresectable Hepatocellular Carcinoma: Meta-Analysis of Randomized Controlled Trials. Radiology (2002) 224(1):47–54. doi: 10.1148/radiol.2241011262 12091661

[23] Kim BK Kim SU Kim KA Chung YE Kim MJ Park MS. Complete Response at First Chemoembolization Is Still the Most Robust Predictor for Favorable Outcome in Hepatocellular Carcinoma. J Hepatol (2015) 62(6):1304–10. doi: 10.1016/j.jhep.2015.01.022 25637785

[24] Zhang Y Zhang M Chen M Mei J Xu L Guo R. Association of Sustained Response Duration With Survival After Conventional Transarterial Chemoembolization in Patients With Hepatocellular Carcinoma. JAMA Netw Open (2018) 1(6):e183213. doi: 10.1001/jamanetworkopen.2018.3213 30646226 PMC6324454

[25] Bruix J Sala M Llovet JM. Chemoembolization for Hepatocellular Carcinoma. Gastroenterology (2004) 127(5 Suppl 1):S179–88. doi: 10.1053/j.gastro.2004.09.032 15508083

[26] Facciorusso A Licinio R Muscatiello N Di Leo A Barone M. Transarterial Chemoembolization: Evidences From the Literature and Applications in Hepatocellular Carcinoma Patients. World J Hepatol (2015) 7(16):2009–19. doi: 10.4254/wjh.v7.i16.2009 PMC4528274 26261690

[27] de Baere T Arai Y Lencioni R Geschwind JF Rilling W Salem R. Treatment of Liver Tumors With Lipiodol TACE: Technical Recommendations From Experts Opinion. Cardiovasc Intervent Radiol (2016) 39(3):334–43. doi: 10.1007/s00270-015-1208-y 26390875

[28] Melchiorre F Patella F Pescatori L Pesapane F Fumarola E Biondetti P. DEB-TACE: A Standard Review. Future Oncol (London England) (2018) 14(28):2969–84. doi: 10.2217/fon-2018-0136 29987957

[29] Facciorusso A. Drug-Eluting Beads Transarterial Chemoembolization for Hepatocellular Carcinoma: Current State of the Art. World J Gastroenterol (2018) 24(2):161–9. doi: 10.3748/wjg.v24.i2.161 PMC5768935 29375202

[30] Hong K Khwaja A Liapi E Torbenson MS Georgiades CS Geschwind JF. New Intra-Arterial Drug Delivery System for the Treatment of Liver Cancer: Preclinical Assessment in a Rabbit Model of Liver Cancer. Clin Cancer Res (2006) 12(8):2563–7. doi: 10.1158/1078-0432.Ccr-05-2225 16638866

[31] Lewis AL Gonzalez MV Lloyd AW Hall B Tang Y Willis SL. DC Bead: *In Vitro* Characterization of a Drug-Delivery Device for Transarterial Chemoembolization. J Vasc Intervent Radiol: JVIR (2006) 17(2 Pt 1):335–42. doi: 10.1097/01.Rvi.0000195323.46152.B3 16517780

[32] Poon RT Tso WK Pang RW Ng KK Woo R Tai KS. A Phase I/II Trial of Chemoembolization for Hepatocellular Carcinoma Using a Novel Intra-Arterial Drug-Eluting Bead. Clin Gastroenterol Hepatol (2007) 5(9):1100–8. doi: 10.1016/j.cgh.2007.04.021 17627902

[33] Schicho A Hellerbrand C Krüger K Beyer LP Wohlgemuth W Niessen C. Impact of Different Embolic Agents for Transarterial Chemoembolization (TACE) Procedures on Systemic Vascular Endothelial Growth Factor (VEGF) Levels. J Clin Transl Hepatol (2016) 4(4):288–92. doi: 10.14218/jcth.2016.00058 PMC5225147 28097096

[34] Lammer J Malagari K Vogl T Pilleul F Denys A Watkinson A. Prospective Randomized Study of Doxorubicin-Eluting-Bead Embolization in the Treatment of Hepatocellular Carcinoma: Results of the PRECISION V Study. Cardiovasc Intervent Radiol (2010) 33(1):41–52. doi: 10.1007/s00270-009-9711-7 19908093 PMC2816794

[35] Golfieri R Giampalma E Renzulli M Cioni R Bargellini I Bartolozzi C. Randomised Controlled Trial of Doxorubicin-Eluting Beads vs Conventional Chemoembolisation for Hepatocellular Carcinoma. Br J Cancer (2014) 111(2):255–64. doi: 10.1038/bjc.2014.199 PMC4102934 24937669

[36] Kloeckner R Weinmann A Prinz F Pinto dos Santos D Ruckes C Dueber C. Conventional Transarterial Chemoembolization Versus Drug-Eluting Bead Transarterial Chemoembolization for the Treatment of Hepatocellular Carcinoma. BMC Cancer (2015) 15:465. doi: 10.1186/s12885-015-1480-x 26059447 PMC4460638

[37] Karalli A Teiler J Haji M Seth E Brismar TB Wahlin S. Comparison of Lipiodol Infusion and Drug-Eluting Beads Transarterial Chemoembolization of Hepatocellular Carcinoma in a Real-Life Setting. Scand J Gastroenterol (2019) 54(7):905–12. doi: 10.1080/00365521.2019.1632925 31287338

[38] Zhang L Sun JH Ji JS Zhong BY Zhou GH Song JJ. Imaging Changes and Clinical Complications After Drug-Eluting Beads Versus Conventional Transarterial Chemoembolization for Unresectable Hepatocellular Carcinoma: Multicenter Study. AJR Am J Roentgenol (2020) 217(4):933-43. doi: 10.2214/ajr.20.24708 33245680

[39] Shiozawa K Matsui T Murakami T Watanabe M Maetani I. Predicting Therapeutic Efficacy of Transarterial Chemoembolization With Drug-Eluting Beads for Hepatocellular Carcinoma Using Contrast-Enhanced Ultrasound. Diagn (Basel Switzerland) (2021) 11(2):291. doi: 10.3390/diagnostics11020291 PMC7917690 33673221

[40] Lee KH Liapi E Vossen JA Buijs M Ventura VP Georgiades C. Distribution of Iron Oxide-Containing Embosphere Particles After Transcatheter Arterial Embolization in an Animal Model of Liver Cancer: Evaluation With MR Imaging and Implication for Therapy. J Vasc Intervent Radiol: JVIR (2008) 19(10):1490–6. doi: 10.1016/j.jvir.2008.06.008 PMC2747287 18755602

[41] Yamamoto A Imai S Kobatake M Yamashita T Tamada T Umetani K. Evaluation of Tris-Acryl Gelatin Microsphere Embolization With Monochromatic X Rays: Comparison With Polyvinyl Alcohol Particles. J Vasc Intervent Radiol: JVIR (2006) 17(11 Pt 1):1797–802. doi: 10.1097/01.RVI.0000243614.87529.b0 17142710

[42] Padia SA Shivaram G Bastawrous S Bhargava P Vo NJ Vaidya S. Safety and Efficacy of Drug-Eluting Bead Chemoembolization for Hepatocellular Carcinoma: Comparison of Small-Versus Medium-Size Particles. J Vasc Intervent Radiol: JVIR (2013) 24(3):301–6. doi: 10.1016/j.jvir.2012.11.023 23380737

[43] Huo YR Xiang H Chan MV Chan C. Survival, Tumour Response and Safety of 70-150 μm Versus 100-300 μm Doxorubicin Drug-Eluting Beads in Transarterial Chemoembolisation for Hepatocellular Carcinoma. J Med Imaging Radiat Oncol (2019) 63(6):802–11. doi: 10.1111/1754-9485.12971 31709778

[44] Urbano J Echevarria-Uraga JJ Ciampi-Dopazo JJ Sánchez-Corral JA Cobos Alonso J Anton-Ladislao A. Multicentre Prospective Study of Drug-Eluting Bead Chemoembolisation Safety Using Tightly Calibrated Small Microspheres in Non-Resectable Hepatocellular Carcinoma. Eur J Radiol (2020) 126:108966. doi: 10.1016/j.ejrad.2020.108966 32278280

[45] Malagari K Moschouris H Kiakidis T Harward S Kelekis A Vrakas S. Five-Years Outcome Analysis of 142 Consecutive Hepatocellular Carcinoma Patients Treated With Doxorubicin Eluting Microspheres 30-60 μm: Results From a Single-Centre Prospective Phase II Trial. Cardiovasc Intervent Radiol (2019) 42(11):1551–62. doi: 10.1007/s00270-019-02260-3 31321482

[46] Ballı HT Aikimbaev K. Super-Selective Transarterial Chemoembolization of Hepatocellular Carcinoma With Doxorubicin-Eluting Beads Sized 40-75 Microns: Assessment of Efficacy and Safety. Diagn Intervent Radiol (Ankara Turkey) (2020) 26(5):482–7. doi: 10.5152/dir.2020.19410 PMC7490015 32815520

[47] Chang WC Hsu HH Chiu SH Huang WY Lo CH Lin HH. Transcatheter Arterial Chemoembolization With Drug-Eluting Beads for the Treatment of Hepatocellular Carcinoma: Recommended Selection for Small-Caliber (<100 μm) Beads. J Hepatocell Carcinoma (2021) 8:937–49. doi: 10.2147/jhc.S319920 PMC8373306 34422707

[48] Sun HC Tang ZY. Angiogenesis in Hepatocellular Carcinoma: The Retrospectives and Perspectives. J Cancer Res Clin Oncol (2004) 130(6):307–19. doi: 10.1007/s00432-003-0530-y PMC12161861 15034787

[49] Qin S Li A Yi M Yu S Zhang M Wu K. Recent Advances on Anti-Angiogenesis Receptor Tyrosine Kinase Inhibitors in Cancer Therapy. J Hematol Oncol (2019) 12(1):27. doi: 10.1186/s13045-019-0718-5 30866992 PMC6417086

[50] Riedl CC Brader P Zanzonico PB Chun YS Woo Y Singh P. Imaging Hypoxia in Orthotopic Rat Liver Tumors With Iodine 124-Labeled Iodoazomycin Galactopyranoside PET. Radiology (2008) 248(2):561–70. doi: 10.1148/radiol.2482071421 PMC2797648 18641253

[51] McKeown SR. Defining Normoxia, Physoxia and Hypoxia in Tumours-Implications for Treatment Response. Br J Radiol (2014) 87(1035):20130676. doi: 10.1259/bjr.20130676 24588669 PMC4064601

[52] Xiong XX Qiu XY Hu DX Chen XQ. Advances in Hypoxia-Mediated Mechanisms in Hepatocellular Carcinoma. Mol Pharmacol (2017) 92(3):246–55. doi: 10.1124/mol.116.107706 28242743

[53] Wada H Nagano H Yamamoto H Yang Y Kondo M Ota H. Expression Pattern of Angiogenic Factors and Prognosis After Hepatic Resection in Hepatocellular Carcinoma: Importance of Angiopoietin-2 and Hypoxia-Induced Factor-1 Alpha. Liver Int (2006) 26(4):414–23. doi: 10.1111/j.1478-3231.2006.01243.x 16629644

[54] Jain RK Tong RT Munn LL. Effect of Vascular Normalization by Antiangiogenic Therapy on Interstitial Hypertension, Peritumor Edema, and Lymphatic Metastasis: Insights From a Mathematical Model. Cancer Res (2007) 67(6):2729–35. doi: 10.1158/0008-5472.Can-06-4102 PMC3022341 17363594

[55] Zhu AX Duda DG Sahani DV Jain RK. HCC and Angiogenesis: Possible Targets and Future Directions. Nat Rev Clin Oncol (2011) 8(5):292–301. doi: 10.1038/nrclinonc.2011.30 21386818 PMC3266719

[56] Lee A Lee FC. Medical Oncology Management of Advanced Hepatocellular Carcinoma 2019: A Reality Check. Front Med (2020) 14(3):273–83. doi: 10.1007/s11684-019-0728-2 31863306

[57] Llovet JM Ricci S Mazzaferro V Hilgard P Gane E Blanc JF. Sorafenib in Advanced Hepatocellular Carcinoma. N Engl J Med (2008) 359(4):378–90. doi: 10.1056/NEJMoa0708857 18650514

[58] Piscaglia F Ogasawara S. Patient Selection for Transarterial Chemoembolization in Hepatocellular Carcinoma: Importance of Benefit/Risk Assessment. Liver Cancer (2018) 7(1):104–19. doi: 10.1159/000485471 PMC5892363 29662837

[59] Kudo M Ueshima K Chan S Minami T Chishina H Aoki T. Lenvatinib as an Initial Treatment in Patients With Intermediate-Stage Hepatocellular Carcinoma Beyond Up-To-Seven Criteria and Child-Pugh A Liver Function: A Proof-Of-Concept Study. Cancers (2019) 11(8). doi: 10.3390/cancers11081084 PMC6721438 31370183

[60] Lo CM Ngan H Tso WK Liu CL Lam CM Poon RT. Randomized Controlled Trial of Transarterial Lipiodol Chemoembolization for Unresectable Hepatocellular Carcinoma. Hepatol (Baltimore Md) (2002) 35(5):1164–71. doi: 10.1053/jhep.2002.33156 11981766

[61] Hiraoka A Kumada T Kudo M Hirooka M Koizumi Y Hiasa Y. Hepatic Function During Repeated TACE Procedures and Prognosis After Introducing Sorafenib in Patients With Unresectable Hepatocellular Carcinoma: Multicenter Analysis. Digest Dis (Basel Switzerland) (2017) 35(6):602–10. doi: 10.1159/000480256 29040999

[62] Raoul JL Sangro B Forner A Mazzaferro V Piscaglia F Bolondi L. Evolving Strategies for the Management of Intermediate-Stage Hepatocellular Carcinoma: Available Evidence and Expert Opinion on the Use of Transarterial Chemoembolization. Cancer Treat Rev (2011) 37(3):212–20. doi: 10.1016/j.ctrv.2010.07.006 20724077

[63] Bolondi L Burroughs A Dufour JF Galle PR Mazzaferro V Piscaglia F. Heterogeneity of Patients With Intermediate (BCLC B) Hepatocellular Carcinoma: Proposal for a Subclassification to Facilitate Treatment Decisions. Semin Liver Dis (2012) 32(4):348–59. doi: 10.1055/s-0032-1329906 23397536

[64] Ha Y Shim JH Kim SO Kim KM Lim YS Lee HC. Clinical Appraisal of the Recently Proposed Barcelona Clinic Liver Cancer Stage B Subclassification by Survival Analysis. J Gastroenterol Hepatol (2014) 29(4):787–93. doi: 10.1111/jgh.12452 24224567

[65] Kudo M Arizumi T Ueshima K Sakurai T Kitano M Nishida N. Subclassification of BCLC B Stage Hepatocellular Carcinoma and Treatment Strategies: Proposal of Modified Bolondi’s Subclassification (Kinki Criteria). Digest Dis (Basel Switzerland) (2015) 33(6):751–8. doi: 10.1159/000439290 26488473

[66] Arizumi T Ueshima K Iwanishi M Minami T Chishina H Kono M. Validation of Kinki Criteria, a Modified Substaging System, in Patients With Intermediate Stage Hepatocellular Carcinoma. Digest Dis (Basel Switzerland) (2016) 34(6):671–8. doi: 10.1159/000448834 27750236

[67] Hung YW Lee IC Chi CT Lee RC Liu CA Chiu NC. Redefining Tumor Burden in Patients With Intermediate-Stage Hepatocellular Carcinoma: The Seven-Eleven Criteria. Liver Cancer (2021) 10(6):629–40. doi: 10.1159/000517393 PMC8647089 34950185

[68] Yamakado K Miyayama S Hirota S Mizunuma K Nakamura K Inaba Y. Prognosis of Patients With Intermediate-Stage Hepatocellular Carcinomas Based on the Child-Pugh Score: Subclassifying the Intermediate Stage (Barcelona Clinic Liver Cancer Stage B). Jpn J Radiol (2014) 32(11):644–9. doi: 10.1007/s11604-014-0358-1 25213426

[69] Yamakado K Miyayama S Hirota S Mizunuma K Nakamura K Inaba Y. Subgrouping of Intermediate-Stage (BCLC Stage B) Hepatocellular Carcinoma Based on Tumor Number and Size and Child-Pugh Grade Correlated With Prognosis After Transarterial Chemoembolization. Jpn J Radiol (2014) 32(5):260–5. doi: 10.1007/s11604-014-0298-9 24615165

[70] Kudo M. Heterogeneity and Subclassification of Barcelona Clinic Liver Cancer Stage B. Liver Cancer (2016) 5(2):91–6. doi: 10.1159/000367768 PMC4906422 27386427

[71] Hiraoka A Kumada T Nouso K Tsuji K Itobayashi E Hirooka M. Proposed New Sub-Grouping for Intermediate-Stage Hepatocellular Carcinoma Using Albumin-Bilirubin Grade. Oncology (2016) 91(3):153–61. doi: 10.1159/000447061 27362669

[72] Hu KS Tang B Yuan J Lu SX Li M Chen RX. A New Substage Classification Strategy for Barcelona Clinic Liver Cancer Stage B Patients With Hepatocellular Carcinoma. J Gastroenterol Hepatol (2019) 34(11):1984–91. doi: 10.1111/jgh.14673 30932246

[73] Kim JH Shim JH Lee HC Sung KB Ko HK Ko GY. New Intermediate-Stage Subclassification for Patients With Hepatocellular Carcinoma Treated With Transarterial Chemoembolization. Liver Int (2017) 37(12):1861–8. doi: 10.1111/liv.13487 28581250

[74] Carmeliet P Jain RK. Angiogenesis in Cancer and Other Diseases. Nature (2000) 407(6801):249–57. doi: 10.1038/35025220 11001068

[75] Kudo M Han G Finn RS Poon RT Blanc JF Yan L. Brivanib as Adjuvant Therapy to Transarterial Chemoembolization in Patients With Hepatocellular Carcinoma: A Randomized Phase III Trial. Hepatol (Baltimore Md) (2014) 60(5):1697–707. doi: 10.1002/hep.27290 24996197

[76] Lencioni R Llovet JM Han G Tak WY Yang J Guglielmi A. Sorafenib or Placebo Plus TACE With Doxorubicin-Eluting Beads for Intermediate Stage HCC: The SPACE Trial. J Hepatol (2016) 64(5):1090–8. doi: 10.1016/j.jhep.2016.01.012 26809111

[77] Meyer T Fox R Ma YT Ross PJ James MW Sturgess R. Sorafenib in Combination With Transarterial Chemoembolisation in Patients With Unresectable Hepatocellular Carcinoma (TACE 2): A Randomised Placebo-Controlled, Double-Blind, Phase 3 Trial. Lancet Gastroenterol Hepatol (2017) 2(8):565–75. doi: 10.1016/s2468-1253(17)30156-5 28648803

[78] Kudo M Cheng A-L Park J-W Park JH Liang P-C Hidaka H. Orantinib Versus Placebo Combined With Transcatheter Arterial Chemoembolisation in Patients With Unresectable Hepatocellular Carcinoma (ORIENTAL): A Randomised, Double-Blind, Placebo-Controlled, Multicentre, Phase 3 Study. Lancet Gastroenterol Hepatol (2018) 3(1):37–46. doi: 10.1016/s2468-1253(17)30290-x 28988687

[79] Kudo M Ueshima K Ikeda M Torimura T Tanabe N Aikata H. Randomised, Multicentre Prospective Trial of Transarterial Chemoembolisation (TACE) Plus Sorafenib as Compared With TACE Alone in Patients With Hepatocellular Carcinoma: TACTICS Trial. Gut (2020) 69(8):1492–501. doi: 10.1136/gutjnl-2019-318934 PMC7398460 31801872

[80] Guo W Chen S Wu Z Zhuang W Yang J. Efficacy and Safety of Transarterial Chemoembolization Combined With Anlotinib for Unresectable Hepatocellular Carcinoma: A Retrospective Study. Technol Cancer Res Treat (2020) 19:1533033820965587. doi: 10.1177/1533033820965587 33089769 PMC7586029

[81] Kudo M Arizumi T. Transarterial Chemoembolization in Combination With a Molecular Targeted Agent: Lessons Learned From Negative Trials (Post-TACE, BRISK-TA, SPACE, ORIENTAL, and TACE-2). Oncology (2017) 93(Suppl 1):127–34. doi: 10.1159/000481243 29258086

[82] Kudo M ed. TACTICS: Final Overall Survival (OS) Data From a Randomized, Open Label, Multicenter, Phase II Trial of Transcatheter Arterial Chemoembolization (TACE) Therapy in Combination With Sorafenib as Compared With TACE Alone in Patients (Pts) With Hepatocellular Carcinoma (HCC). J Clin Oncol (2021) 39(3_suppl):270. doi: 10.1200/JCO.2021.39.3_suppl.270

[83] Llovet JM Montal R Villanueva A. Randomized Trials and Endpoints in Advanced HCC: Role of PFS as a Surrogate of Survival. J Hepatol (2019) 70(6):1262–77. doi: 10.1016/j.jhep.2019.01.028 PMC12452112 30943423

[84] Llovet JM Villanueva A Marrero JA Schwartz M Meyer T Galle PR. Trial Design and Endpoints in Hepatocellular Carcinoma: AASLD Consensus Conference. Hepatol (2021) 73(Suppl 1):158-91. doi: 10.1002/hep.31327 32430997

[85] Park JW Finn RS Kim JS Karwal M Li RK Ismail F. Phase II, Open-Label Study of Brivanib as First-Line Therapy in Patients With Advanced Hepatocellular Carcinoma. Clin Cancer Res (2011) 17(7):1973–83. doi: 10.1158/1078-0432.Ccr-10-2011 21349999

[86] Solorzano CC Jung YD Bucana CD McConkey DJ Gallick GE McMahon G. *In Vivo* Intracellular Signaling as a Marker of Antiangiogenic Activity. Cancer Res (2001) 61(19):7048–51. 11585733

[87] Kuenen BC Giaccone G Ruijter R Kok A Schalkwijk C Hoekman K. Dose-Finding Study of the Multitargeted Tyrosine Kinase Inhibitor SU6668 in Patients With Advanced Malignancies. Clin Cancer Res (2005) 11(17):6240–6. doi: 10.1158/1078-0432.Ccr-04-2466 16144927

[88] Inaba Y Kanai F Aramaki T Yamamoto T Tanaka T Yamakado K. A Randomised Phase II Study of TSU-68 in Patients With Hepatocellular Carcinoma Treated by Transarterial Chemoembolisation. Eur J Cancer (Oxford England: 1990) (2013) 49(13):2832–40. doi: 10.1016/j.ejca.2013.05.011 23764238

[89] Han B Li K Wang Q Zhang L Shi J Wang Z. Effect of Anlotinib as a Third-Line or Further Treatment on Overall Survival of Patients With Advanced Non-Small Cell Lung Cancer: The ALTER 0303 Phase 3 Randomized Clinical Trial. JAMA Oncol (2018) 4(11):1569–75. doi: 10.1001/jamaoncol.2018.3039 PMC6248083 30098152

[90] He C Wu T Hao Y. Anlotinib Induces Hepatocellular Carcinoma Apoptosis and Inhibits Proliferation *via* Erk and Akt Pathway. Biochem Biophys Res Commun (2018) 503(4):3093–9. doi: 10.1016/j.bbrc.2018.08.098 30146257

[91] Zhou M Chen X Zhang H Xia L Tong X Zou L. China National Medical Products Administration Approval Summary: Anlotinib for the Treatment of Advanced Non-Small Cell Lung Cancer After Two Lines of Chemotherapy. Cancer Commun (London England) (2019) 39(1):36. doi: 10.1186/s40880-019-0383-7 PMC6585030 31221221

[92] Shen G Zheng F Ren D Du F Dong Q Wang Z. Anlotinib: A Novel Multi-Targeting Tyrosine Kinase Inhibitor in Clinical Development. J Hematol Oncol (2018) 11(1):120. doi: 10.1186/s13045-018-0664-7 30231931 PMC6146601

[93] Matsuki M Hoshi T Yamamoto Y Ikemori-Kawada M Minoshima Y Funahashi Y. Lenvatinib Inhibits Angiogenesis and Tumor Fibroblast Growth Factor Signaling Pathways in Human Hepatocellular Carcinoma Models. Cancer Med (2018) 7(6):2641–53. doi: 10.1002/cam4.1517 PMC6010799 29733511

[94] Matsui J Funahashi Y Uenaka T Watanabe T Tsuruoka A Asada M. Multi-Kinase Inhibitor E7080 Suppresses Lymph Node and Lung Metastases of Human Mammary Breast Tumor MDA-MB-231 *via* Inhibition of Vascular Endothelial Growth Factor-Receptor (VEGF-R) 2 and VEGF-R3 Kinase. Clin Cancer Res (2008) 14(17):5459–65. doi: 10.1158/1078-0432.Ccr-07-5270 18765537

[95] Matsui J Yamamoto Y Funahashi Y Tsuruoka A Watanabe T Wakabayashi T. E7080, a Novel Inhibitor That Targets Multiple Kinases, Has Potent Antitumor Activities Against Stem Cell Factor Producing Human Small Cell Lung Cancer H146, Based on Angiogenesis Inhibition. Int J Cancer (2008) 122(3):664–71. doi: 10.1002/ijc.23131 17943726

[96] Yamada K Yamamoto N Yamada Y Nokihara H Fujiwara Y Hirata T. Phase I Dose-Escalation Study and Biomarker Analysis of E7080 in Patients With Advanced Solid Tumors. Clin Cancer Res (2011) 17(8):2528–37. doi: 10.1158/1078-0432.Ccr-10-2638 21372218

[97] Boss DS Glen H Beijnen JH Keesen M Morrison R Tait B. A Phase I Study of E7080, a Multitargeted Tyrosine Kinase Inhibitor, in Patients With Advanced Solid Tumours. Br J Cancer (2012) 106(10):1598–604. doi: 10.1038/bjc.2012.154 PMC3349182 22516948

[98] Kudo M Finn RS Qin S Han KH Ikeda K Piscaglia F. Lenvatinib Versus Sorafenib in First-Line Treatment of Patients With Unresectable Hepatocellular Carcinoma: A Randomised Phase 3 Non-Inferiority Trial. Lancet (London England) (2018) 391(10126):1163–73. doi: 10.1016/s0140-6736(18)30207-1 29433850

[99] Fu Z Li X Zhong J Chen X Cao K Ding N. Lenvatinib in Combination With Transarterial Chemoembolization for Treatment of Unresectable Hepatocellular Carcinoma (uHCC): A Retrospective Controlled Study. Hepatol Int (2021) 15(3):663–75. doi: 10.1007/s12072-021-10184-9 PMC8286947 33877527

[100] Finn RS Ryoo BY Merle P Kudo M Bouattour M Lim HY. Pembrolizumab As Second-Line Therapy in Patients With Advanced Hepatocellular Carcinoma in KEYNOTE-240: A Randomized, Double-Blind, Phase III Trial. J Clin Oncol (2020) 38(3):193–202. doi: 10.1200/jco.19.01307 31790344

[101] Zhu AX Finn RS Edeline J Cattan S Ogasawara S Palmer D. Pembrolizumab in Patients With Advanced Hepatocellular Carcinoma Previously Treated With Sorafenib (KEYNOTE-224): A Non-Randomised, Open-Label Phase 2 Trial. Lancet Oncol (2018) 19(7):940–52. doi: 10.1016/s1470-2045(18)30351-6 29875066

[102] Finn RS Qin S Ikeda M Galle PR Ducreux M Kim TY. Atezolizumab Plus Bevacizumab in Unresectable Hepatocellular Carcinoma. N Engl J Med (2020) 382(20):1894–905. doi: 10.1056/NEJMoa1915745 32402160

[103] Zheng L Fang S Wu F Chen W Chen M Weng Q. Efficacy and Safety of TACE Combined With Sorafenib Plus Immune Checkpoint Inhibitors for the Treatment of Intermediate and Advanced TACE-Refractory Hepatocellular Carcinoma: A Retrospective Study. Front Mol Biosci (2020) 7:609322. doi: 10.3389/fmolb.2020.609322 33521054 PMC7843459

[104] Wang Y Zhou C Liu J Shi Q Huang S Yang C. Increased Liquefactive Necrosis Formation After Transarterial Chemoembolization Combined With Molecular Targeted Agents Plus Immune Checkpoint Inhibitors for Hepatocellular Carcinoma. Cancer Manage Res (2021) 13:6935–41. doi: 10.2147/cmar.S328812 PMC8434848 34522136

[105] Ando Y Kawaoka T Amioka K Naruto K Ogawa Y Yoshikawa Y. Efficacy and Safety of Lenvatinib-Transcatheter Arterial Chemoembolization Sequential Therapy for Patients With Intermediate-Stage Hepatocellular Carcinoma. Oncology (2021) 99(8):507–17. doi: 10.1159/000515865 33946070

